# Meta-analysis of RNA-Seq datasets highlights novel genes/pathways involved in fat deposition in fat-tail of sheep

**DOI:** 10.3389/fvets.2023.1159921

**Published:** 2023-05-12

**Authors:** Seyedeh Fatemeh Hosseini, Mohammad Reza Bakhtiarizadeh, Abdolreza Salehi

**Affiliations:** Department of Animal and Poultry Science, College of Aburaihan, University of Tehran, Tehran, Iran

**Keywords:** adipose tissue, lipid metabolism, comparative transcriptome, gene expression—tools and techniques, RNA-Seq

## Abstract

**Introduction:**

Fat-tail in sheep is considered as an important energy reservoir to provide energy as a survival buffer during harsh challenges. However, fat-tail is losing its importance in modern sheep industry systems and thin-tailed breeds are more desirable. Using comparative transcriptome analysis to compare fat-tail tissue between fat- and thin-tailed sheep breeds provides a valuable approach to study the complex genetic factors associated with fat-tail development. However, transcriptomic studies often suffer from issues with reproducibility, which can be improved by integrating multiple studies based on a meta-analysis.

**Methods:**

Hence, for the first time, an RNA-Seq meta-analysis on sheep fat-tail transcriptomes was performed using six publicly available datasets.

**Results and discussion:**

A total of 500 genes (221 up-regulated, 279 down-regulated) were identified as differentially expressed genes (DEGs). A jackknife sensitivity analysis confirmed the robustness of the DEGs. Moreover, QTL and functional enrichment analysis reinforced the importance of the DEGs in the underlying molecular mechanisms of fat deposition. Protein-protein interactions (PPIs) network analysis revealed the functional interactions among the DEGs and the subsequent sub-network analysis led to identify six functional sub-networks. According to the results of the network analysis, down-regulated DEGs in green and pink sub-networks (like collagen subunits IV, V, and VI, integrins 1 and 2, *SCD, SCD5, ELOVL6, ACLY, SLC27A2*, and *LPIN1*) may impair lipolysis or fatty acid oxidation and cause fat accumulation in tail. On the other hand, up-regulated DEGs, especially those are presented in green and pink sub-networks (like *IL6, RBP4, LEPR, PAI-1, EPHX1, HSD11B1*, and *FMO2*), might contribute to a network controlling fat accumulation in the tail of sheep breed through mediating adipogenesis and fatty acid biosynthesis. Our results highlighted a set of known and novel genes/pathways associated with fat-tail development, which could improve the understanding of molecular mechanisms behind fat deposition in sheep fat-tail.

## Introduction

In recent years, with the growth of world population, there is a considerable demand for the agricultural improvement, especially for meat and milk of animals. In modern agriculture, sheep play an important role in the production of meat, wool and milk. Currently, there are a lot of sheep breeds over a wide geographical range worldwide with a large variation in many phenotypic traits. One of the most important phenotypic traits in sheep is the ability to store of fat in the tail. In this regard, generally sheep breeds can be divided into two main groups (thin- and fat-tailed breeds) and five subgroups including fat-long, fat-short, fat-rumped, thin-long, and thin-short tailed sheep breeds (1). It is well-known that the fat-tailed sheep breeds are evolved from its thin-tailed ancestor (~5,000 years ago), by the principle of selective breeding (natural or artificial selection) and following initial domestication, in response to hazardous environments (2, 3). Fat-tail is considered as an important energy reservoir for the animal to provide energy as a survival buffer during harsh challenges such as drought and food deprivation periods like winter, when pasture is dormant (4). Nowadays, fat-tail is losing its importance and lean-tailed breeds are more desirable, which can be explained by providing three reasons: (1) In modern sheep farming systems, intensive or semi-intensive feeding systems are preferred, which cause fat-tail be no longer important as an energy source; (2) fat deposition led to a higher energetic cost than accretion of an equivalent amount of lean tissue and decreases the feed efficiency (5); and (3) in modern society, consumers prefer low-fat foods to be healthy. Therefore, both customers and producers prefer thin-tailed sheep (6). It is estimated that the fat-tailed sheep breeds constitute about 26% of the world sheep population (7). Hence, study of the fat deposition in sheep tail is of great importance to understand the background molecular mechanisms of the fat-tail development as well as to develop the new breeding strategies to produce improved breeding sheep herds.

Up to now, several transcriptome-based studies have been conducted to elucidate the factors and molecular mechanisms responsible for the differences in fat deposition between different sheep breeds (6, 8–17). Earlier studies were focused on single gene to identify the candidate genes involved in regulating the fat-tail formation. In this regard, higher expression of the leptin (LEP) gene in fat lines of Coopworth sheep was reported for different fat tissues when comparing fat lines and lean lines in Coopworth sheep breed (18). In our previous study, fatty acid binding protein 4 (*FABP4*) gene was suggested as a candidate gene relevant to fat deposition in sheep breeds (8). Moreover, uncoupling protein 1 (*UCP1*) (15) and cell death-inducing DFFA-like effector c (*CIDEC*) (19) genes were reported as other candidate genes involved in regulation of fat deposition and mobilization in tail of sheep breeds.

In recent years, rapid development of next generation sequencing (*NGS*) technologies has lowered the barriers to perform high-throughput gene expression profiling through RNA sequencing (RNA-Seq) approach. This method made it possible to measure the expression of thousands of genes simultaneously, which enables us to better understand the genetic factors associated with phenotypic differences in sheep fat-tail. To date, several studies have been employed this approach to identify the genes linked to fat-tail deposition by conducting a comparative transcriptome study on the fat- and thin-tailed sheep breeds (6, 8–15, 17, 19).

A review of these studies indicates the variability in their results including different set of DEGs or inconsistent gene expression patterns, which can be attributed to different bioinformatics pipeline used, number of biological replicates and other limitations coming from the different nature of the samples. In this context, a meta-analysis of several independent studies focused on a specific biological question, offers a useful approach to increase the statistical power. Additionally, combining evidence from several studies increases the reliability and robustness of the results (6, 11–14, 17, 20). In this study, for the first time a meta-analysis of six independent RNA-Seq studies was performed to provide a basis for the identification of mechanisms underlying fat deposition in sheep tail, which can be exploited in future breeding strategies.

## Materials and methods

### RNA-Seq datasets

**Figure 1:**
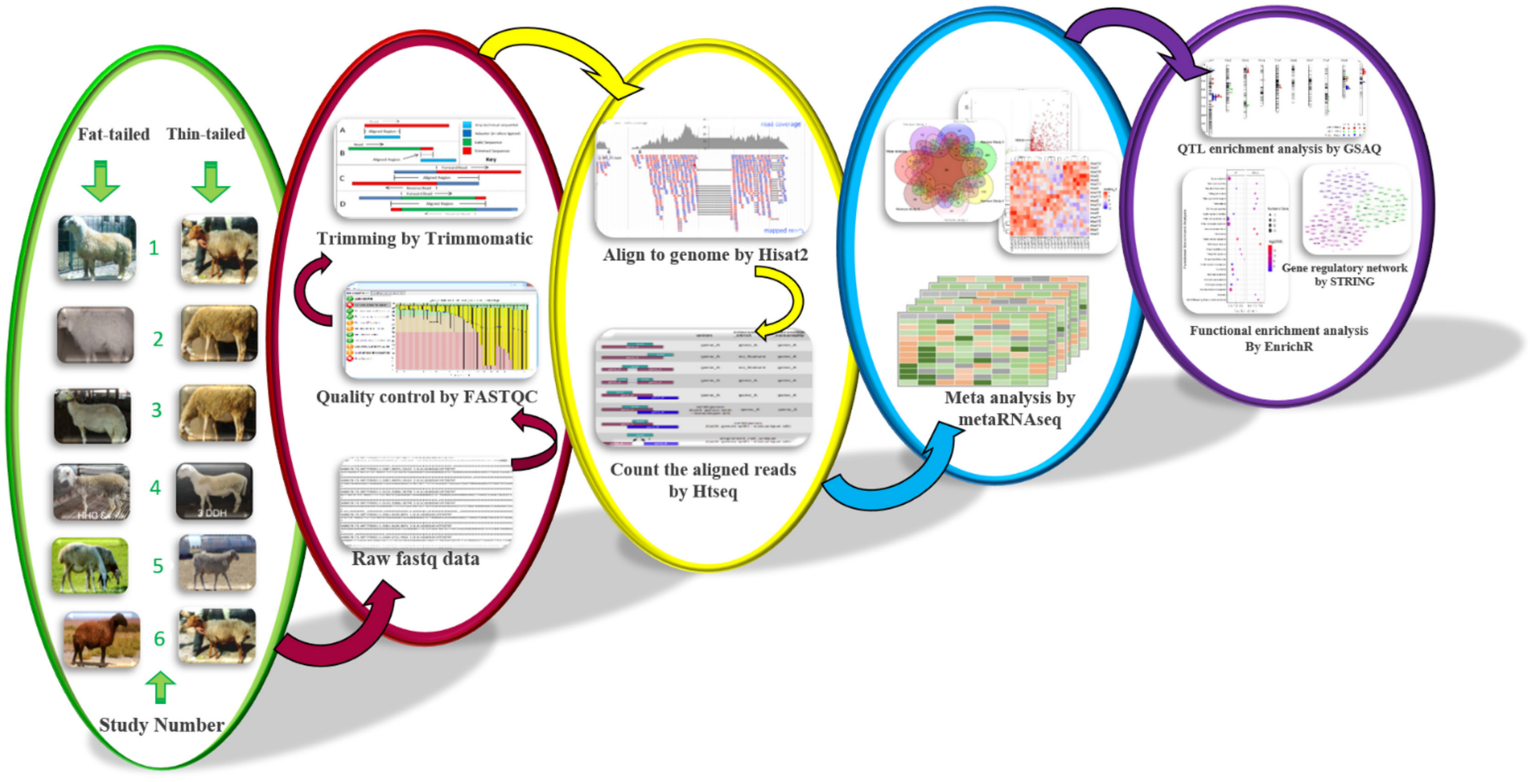
The bioinformatics pipeline for RNA-Seq meta-analysis of fat-tail tissue in sheep breeds.

A comprehensive review of authoritative papers was conducted to find the studies that performed gene expression profiling by RNA-Seq method to identify molecular mechanisms affecting sheep fat-tails (Table 1, Supplementary material 1). Out of 11 identified studies, four studies (11, 13, 14, 17) had one biological replication per breed and data of one study was not available (17) in the public databases. Therefore, six datasets were remained to be used in this study. The samples of these datasets were related to fat-tail tissue of adult male sheep with at least three biological replications per breed (accession numbers of these studies are bolded in Table 1). Only male samples of Fan et al. (9) study were used. RNA-Seq reads for the selected studies (Table 1) were retrieved from the NCBI's sequence read archive database (SRA: https://trace.ncbi.nlm.nih.gov/Traces/sra/). All samples of each study were processed with the same bioinformatic pipeline, explained as follow (Figure 1).

**Table 1:** List of the comparative transcriptome studies on the fat- and thin-tailed sheep breeds.

Fat (Nu of BR)^*^	Thin (Nu of BR)^*^	Up genes^**^	Down genes^**^	Type of reads^***^	Sex^****^	**Accession number**	**References**
Kazak (1)	Tibetan (1)	280	366	P (100)	M	NA	(14)
Dorset (1)	Han (1)	266	336	P (100)	M, F	NA	(13)
Guangling (1)	Han (1)	3,160	2,235	P (90)	M	SRP113440	(11)
Lanzhou (3)	Han (3)	7	3	P (150)	M	**PRJNA432669**	(12)
Lori (3)	Zel (3)	80	184	P (150)	M	**PRJNA508203**	(6)
Hulun-Fat (3)	Hulun-Thin (3)	515	1,012	P (101)	M, F	**PRJNA517348**	(9)
Han-Fat (3)	Han-Thin (3)	237	284	P (75)	M	**PRJNA699984**	(20)
DHH (3)	DDH (3)	262	181	P (150)	M	**PRJNA745517**	(16)
Ghezel (3)	Zel (4)	254	78	P (150)	M	**PRJNA598581**	(10)
Bashby (3)	Bashby-Argali (3)	-	-	P (150)	M	NA	(21)
Altay (1)	XFW (1)	1,389	6,652	P (100)	M	PRJNA627341	(17)

^*^Name of the fat- and thin-tailed breeds (Number of biological replications).

^**^Up- and down-regulated genes in fat-tailed sheep breed.

^***^P, paired-end and S, single-end reads (length of reads).

^****^M, male and F, female. The bolded accession numbers are used in this study. The link address of these data are provided in “Data availability statement” section.

### Differential expression analysis

The quality of RNA-Seq reads was checked using FastQC (v0.11.5) software (22). Trimmomatic (v0.38) software (23) was applied to remove low-quality reads/bases and adaptor sequences based on its adaptive trimming algorithm, maximum information (MAXINFO), to balance the benefits of retaining longer reads against the drawback of having low-quality bases. The other trimming criteria were TRAILING: 20, MAXINFO: 80: 0.8, MINLEN: 80. FastQC was used again to re-evaluate the quality of the clean reads. The clean reads were then aligned to the ENSEMBL ovine reference genome (rambouillet_v1.0.104) using HISAT2 (24) software (version 2.1.0) based on the default parameters. A list of exon-exon junctions extracted from the Ensembl ovine GTF file (rambouillet_v1.0.104) was applied to guide the read mapping. To quantify the aligned reads to annotated genes, python script HTSeq-count (version 2.7.3) tool (25) was used (union intersection mode) according to Ensembl ovine GTF file (rambouillet_v1.0.104) and a gene expression matrix was created for each study. Genes with low read counts (< 10 reads across all samples) were filtered out. DESeq2 (v 1.32.0) was used to normalize and perform differential expression analysis for individual studies using the median-of-ratios method as a normalization factor (26). DESeq2 used Cox-Reid adjusted profile likelihood approach to estimate dispersions (27).

### RNA-Seq meta-analysis

Raw *p*-values obtained from previous step were applied to perform meta-analysis using metaRNAseq R package (v1.0.7) (28). There are two *p*-value combination methods to combine *p*-values from different studies in this package including Fisher's and inverse normal methods. It is reported that both methods provide similar results. Using these methods enable us to combine results from heterogeneous datasets directly to identify commonly regulated genes among all studies. Here, to minimize the false positive results, both methods were used and differentially expressed genes (DEGs) with a false discovery rate (FDR) < 0.05 found in common between two methods were considered statistically significant. One of the limitations of *p*-value-based methods is that the combined *p*-values are estimated regardless of the expression patterns in the different studies. In other words, some genes are significantly up-regulated in some studies, while in the other studies they are significantly down-regulated, which may not reflect the biological reality (28). This limitation can be overcome by selecting the DEGs that showed the same expression pattern in most of studies. In the present study, out of the DEGs that were common between the two methods, only those genes that showed the same expression trend (up or down regulated) in more than half of the datasets, were finally considered as DEGs.

### Jackknife analysis

One of the concerns in meta-analysis is that the identified DEGs can be affected by the properties of a single dataset (29). Hence, to evaluate the robustness of the meta-analysis results, a Jackknife sensitivity analysis was applied (30). To do this, meta-analysis was repeated (as described above) six times, each time including all the datasets except one and six sets of DEGs were generated. Finally, number of presence/absence of DEGs in the six sub meta-analysis was compared with the main meta-analysis.

### Functional enrichment analysis

To gain further insight into the biological processes and pathways significantly enriched in DEGs, functional enrichment analysis was performed. To do this, EnrichR web-based tool was applied, using default parameters, focusing on gene ontology (GO; biological process) and the kyoto gene and genome encyclopedia (KEGG) (31). Also, up- and down-regulated DEGs were submitted separately and FDR < 0.05 was considered for the selection of the significantly enriched GO terms and pathways.

### QTL enrichment analysis

To determine if the DEGs are located in the QTLs associated with fat metabolism, a co-localization analysis was applied. To do this, all QTLs related to fatness were obtained from sheep AnimalQTLdb database (32). Then, enrichment of the DEGs within the QTLs were analyzed using GSAQ R package based on Gene Set Validation with QTL (GSVQ) method (33).

### Network analysis

To reveal the protein-protein interactions (PPIs) among the DEGs, STRING (34) database (v11.5) was applied using *Ovis aries* as the reference organism. The PPIs networks were constructed for up- and down-regulated DEGs, separately. Confidence score < 0.4 (6) was considered to discard unreliable PPIs. Moreover, sub-networks within the networks were identified based on the k-means clustering approach (three clusters) in the database. Cytoscape software (version 3.9) was used to visualize the networks (35).

## Results

### RNA-Seq data analysis

Six RNA-Seq datasets were analyzed including 18 fat-tailed and 19 thin-tailed sheep breeds (Table 1). In total, more than 952 million paired-end raw reads were obtained for the six datasets, ranging from 7.5 to 50.5 million per sample. Among these, 468 and 484 million reads were belonged to fat- and thin-tailed breeds, respectively. Only 1.6 million reads (~0.002%) were removed after quality control and filtering, which shows the quality of the datasets. The clean reads were then aligned to the sheep genome with an average of 87.26% read mapping rate (Table 2 and Supplementary material 1).

**Table 2:** Information of number of reads, alignment rate per sample, and number of DEGs per study.

**Study**	Average Nu of reads ±SD^*^	**Average alignment rate ±SD**	**Nu of DEGs (up and down)**	**References**
Study 1	21.63 ± 2.42	91.10 ± 0.03	602 (224, 378)	(6)
Study 2	17.68 ± 6.66	78.18 ± 0.13	442 (222, 220)	(20)
Study 3	42.73 ± 4.32	74.05 ± 0.04	30 (12, 18)	(12)
Study 4	22.42 ± 2.25	92.81 ± 0.01	60 (16, 44)	(16)
Study 5	27.74 ± 4.37	93.13 ± 0.03	548 (482, 66)	(9)
Study 6	22.720 ± 1.50	93.32 ± 0.02	14 (6, 8)	(10)

^*^Million reads.

### Meta*-*analysis

**Figure 2:**
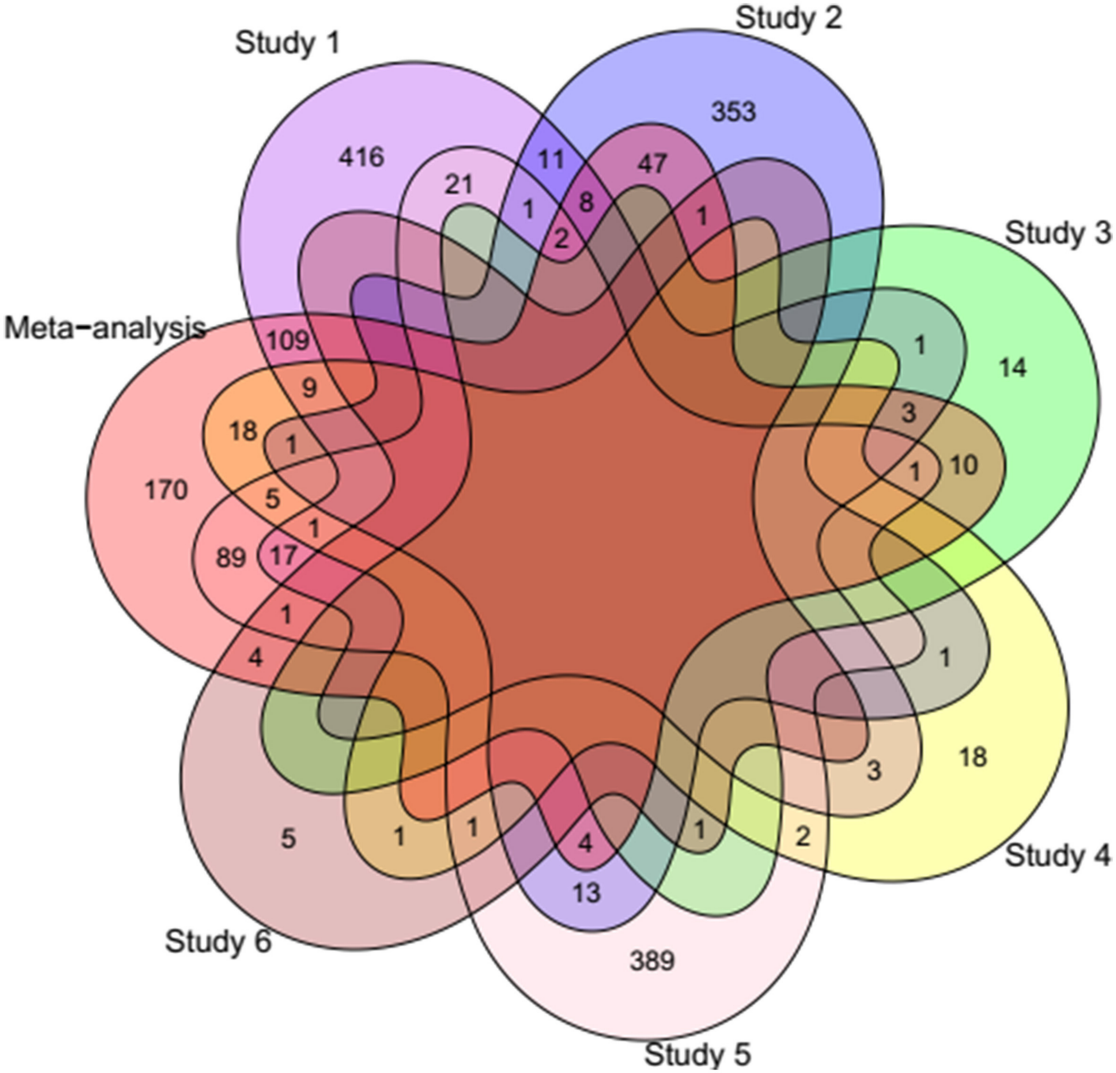
Venn diagram showing the number of common DEGs shared among the individual studies and meta-analysis. Study 1 (6), Study 2 (20), Study 3 (12), Study 4 (16), Study 5 (9), and Study 6 (10).

Differential gene expression analysis was performed for each study and the raw *p*-values of all studies were integrated based on our meta-analysis approach. After multiple testing correction, 761 DEGs were identified. Inspecting the DEGs to have the same log-fold change direction across most of the studies led to identification of 500 DEGs including 221 up- and 279 down-regulated genes in fat- against thin-tailed sheep breeds. Results of the differential expression analysis per study and meta-analysis can be found in Table 2 and Supplementary material 2. To illustrate the common DEGs shared among the individual studies and meta-analysis, a venn diagram was constructed (Figure 2). Out of 500 DEGs, 330 DEGs were shared with the other studies and 170 DEGs were identified only through the meta-analysis. Moreover, most of the identified DEGs from each individual study were not shared with the other studies. No DEG was found to be common among all the studies and meta-analysis.

### Jackknife sensitivity analysis

**Figure 3:**
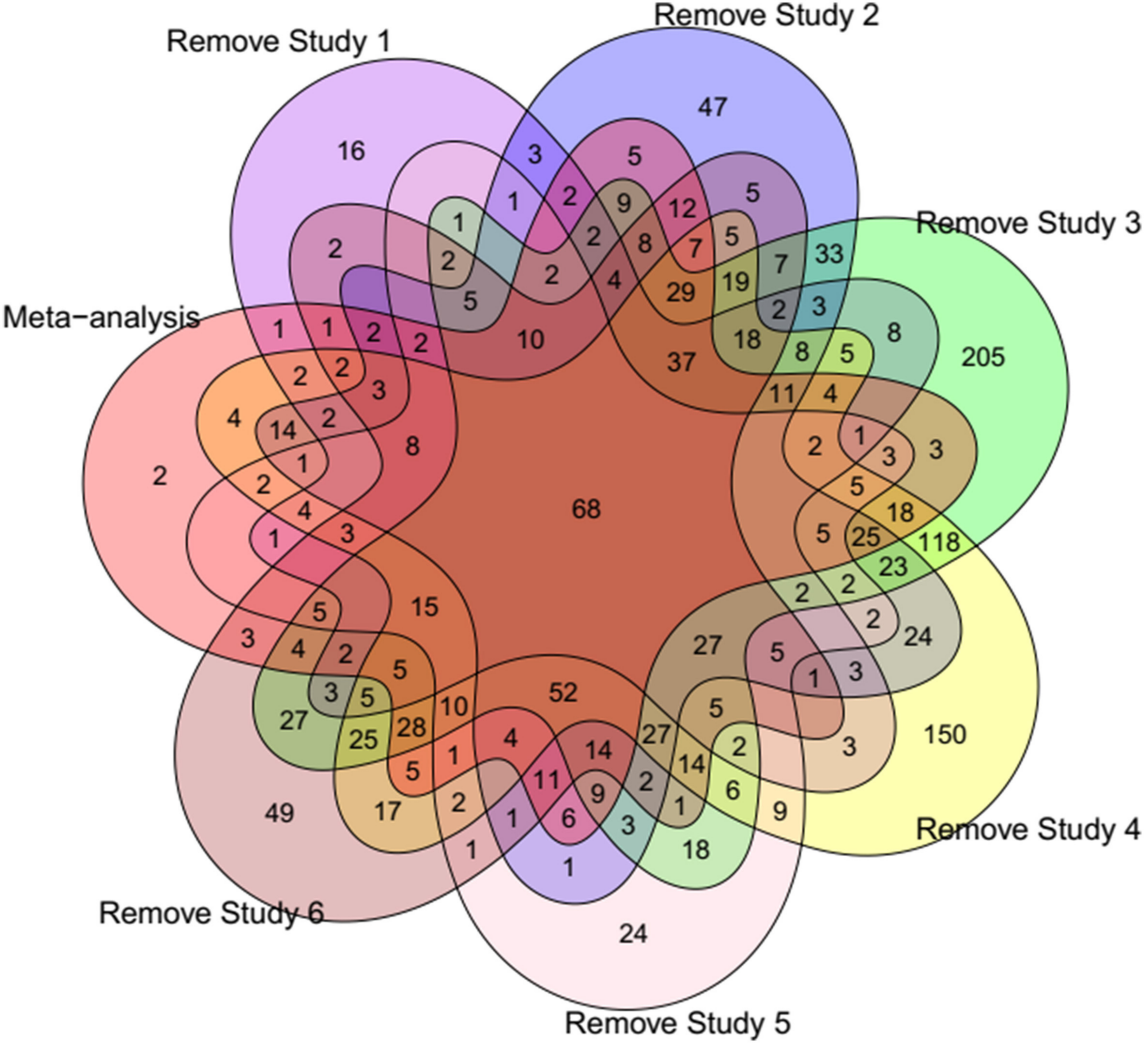
Venn diagram showing the number of common DEGs among different Jackknife tests and meta-analysis results. Study 1 (6), Study 2 (20), Study 3 (12), Study 4 (16), Study 5 (9), and Study 6 (10).

**Figure 4:**
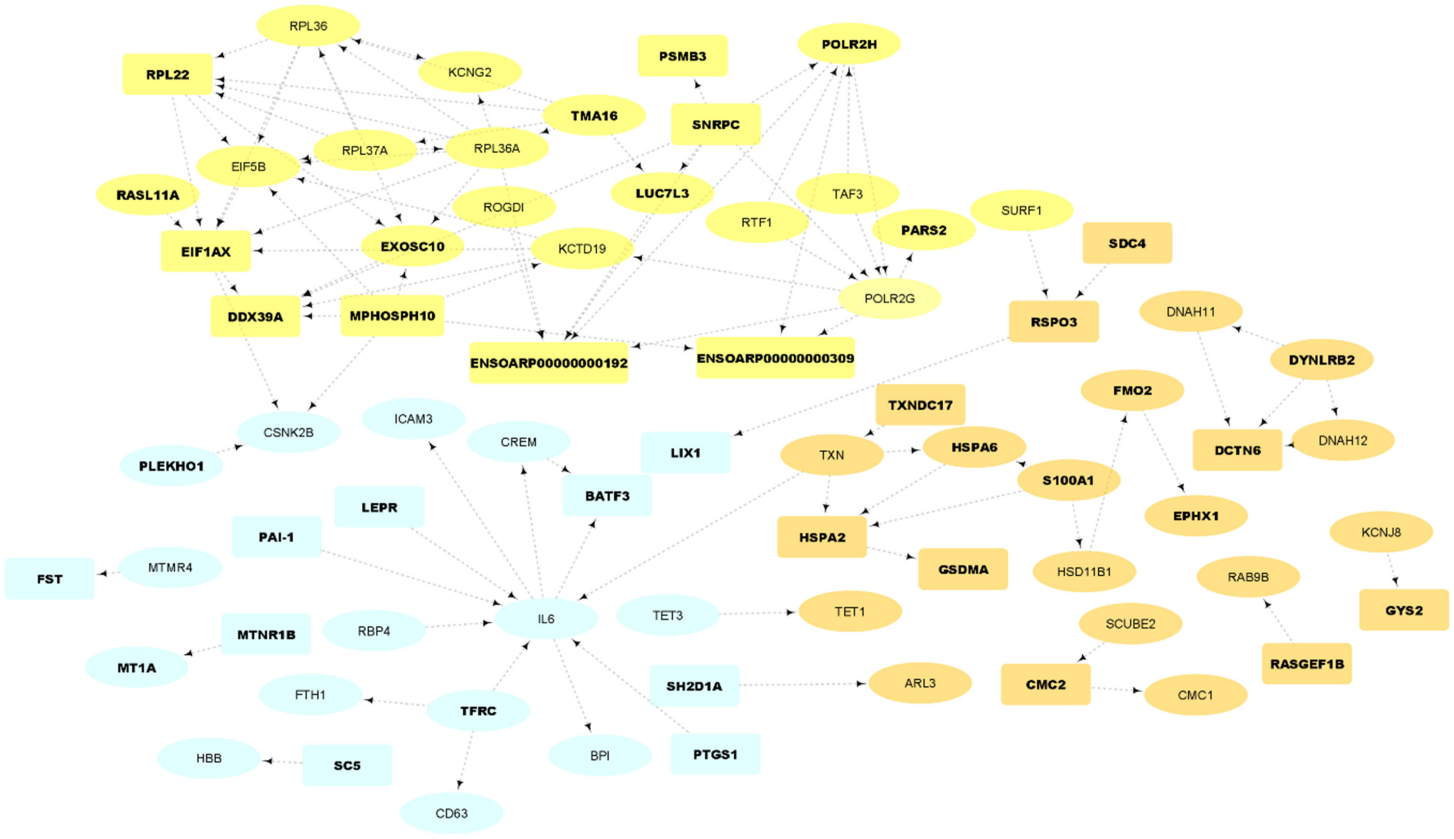
PPI network and functional clusters of the up-regulated DEGs. Orange, yellow and light blue nodes represent orange, yellow and light blue sub-networks, respectively. Oval circles indicate the DEGs that were presented in at least 50% of the Jackknife test results. The DEGs that were located in QTL regions related to fatness are represented as bold texts.

**Figure 5:**
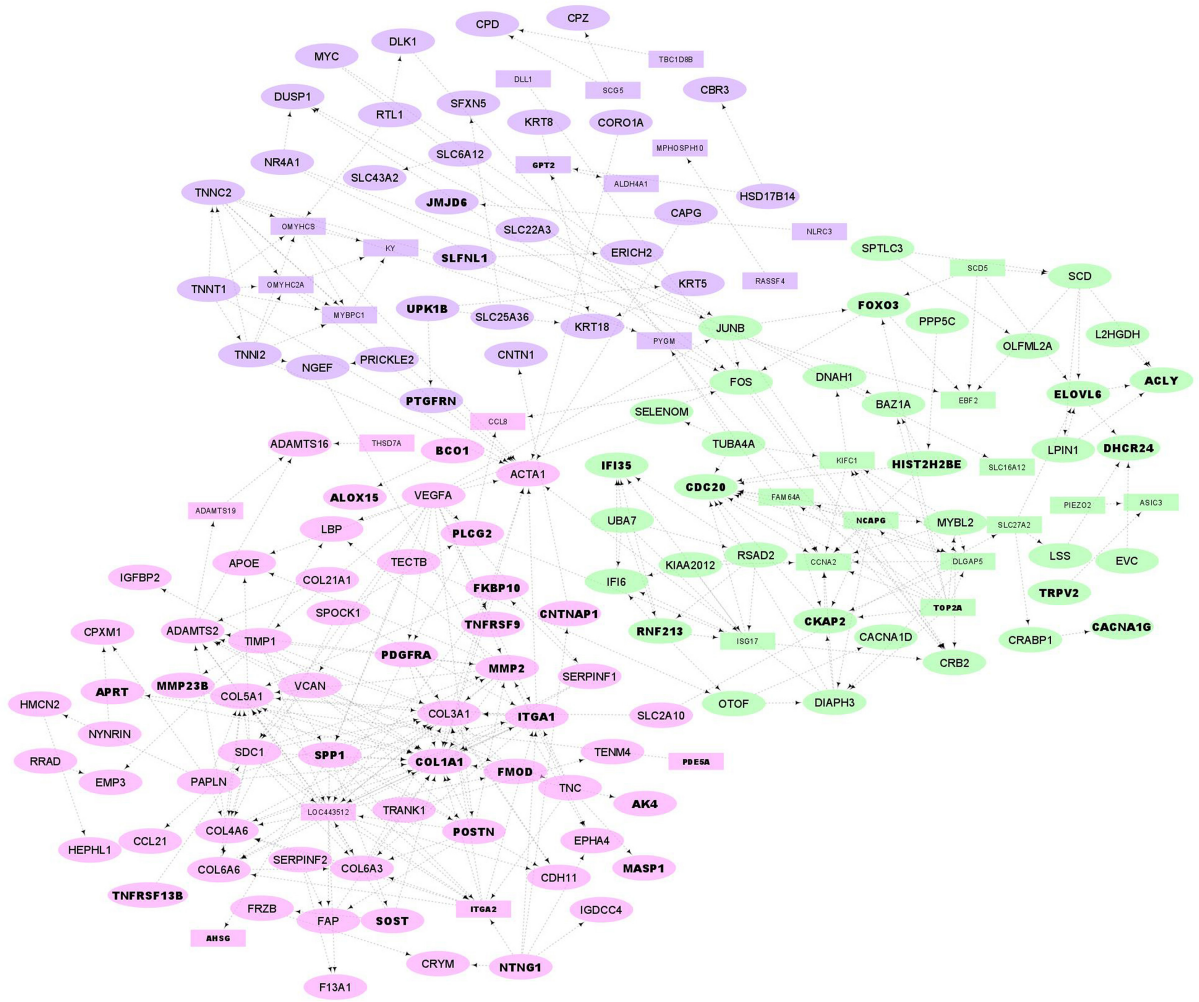
PPI network and functional clusters of the down-regulated DEGs. Green, pink and purple nodes represent green, pink and purple clusters, respectively. Oval circles indicate the DEGs that were presented in at least 50% of the Jackknife test results. The DEGs that were located in QTL regions related to fatness are represented as bold texts.

To confirm that our meta-analysis results were not affected by any single study, a jackknife sensitivity analysis was conducted by iteratively removing one dataset at a time. Figure 3 shows the common DEGs among the six Jackknife tests and the full meta-analysis results. All the identified DEGs by meta-analysis (except two genes *ASIC3* and *MYBPC1*) were identified in at least one Jackknife test. On the other hand, more than 80% of the DEGs (403 genes including 232 down- and 171 up-regulated) were present in at least 50% of the Jackknife test results (oval circles in Figures 4, 5 represent these genes). Sixty-eight DEGs pass all six jackknife analyses and showed complete consistency over the tests, indicative of their importance as they can be considered as a more robust set of potential candidates.

### Functional enrichment analysis

To identify the main biological processes or pathways that the 500 DEGs (including 221 up- and 279 down-regulated genes in fat- against thin-tailed sheep breeds) may be involved in, functional enrichment analysis was performed. In up-regulated genes (higher expression in fat-tailed breeds), none of them were significant at FDR < 0.05 (Supplementary material 3). In total, 26 biological processes and seven KEGG were significantly enriched in down-regulated DEGs (lower expression in fat-tailed breeds) at FDR < 0.05 (Supplementary material 3). It is worthy to highlight the significant GO terms related to fat metabolism including “unsaturated fatty acid biosynthetic process” and “fatty-acyl-CoA biosynthetic process” in the down-regulated DEGs. Thus, it is reasonable to infer that these genes might be related to fat-tail development.

### QTL enrichment analysis

A total of 98 annotated QTLs related to fatness were obtained from the sheep AnimalQTLdb database (32). Totally, 113 genes (including 50 up- and 63 down-regulated as represented in bold text in Figures 4, 5) were significantly (*p*-value = 0.018) located within coordinates of 16 QTLs (Supplementary material 4), which were located in chromosomes 1, 6, 8, 10, 11, 12, 18, 14, 16, 19, and 23. These QTLs pertained to nine different traits including internal fat amount, fat weight in carcass, carcass fat percentage, muscle depth at third lumbar, dressing percentage, subcutaneous fat area, subcutaneous fat thickness, carcass length and lean meat yield percentage. Out of 113 genes, 35 up- and 55 down-regulated DEGs were present in at least 50% of the Jackknife test results (~80%). To check whether the identified DEGs per individual study are significantly enriched in the QTLs, this analysis was performed on the DEGs of each study, separately. Out of six study, only DEGs of the study one was significantly enriched in the QTLs.

### PPI network analysis

To construct an PPI network of DEGs (500 DEGs including 221 up- and 279 down-regulated genes in fat- against thin-tailed sheep breeds), where the nodes are proteins and the edges represent the predicted functional associations, STRING database (34) was used. ENSEMBL accession numbers of DEGs were first annotated and 361 gene names (135 up and 226 down) were found. Of these, 125 and 218 genes were matched with the database and applied to construct the PPI network in up- and down-regulated DEGs, respectively. A total of 72 nodes with 98 edges (known or predicted interactions) composed the final PPIs network of up-regulated DEGs (PPI enrichment *p*-value = 0.002, Figure 4). Further, the gene network interaction of down-regulated DEGs included 157 nodes with 337 edges (PPI enrichment *p*-value = < 1.0e−16, Figure 4).

**Figure 6:**
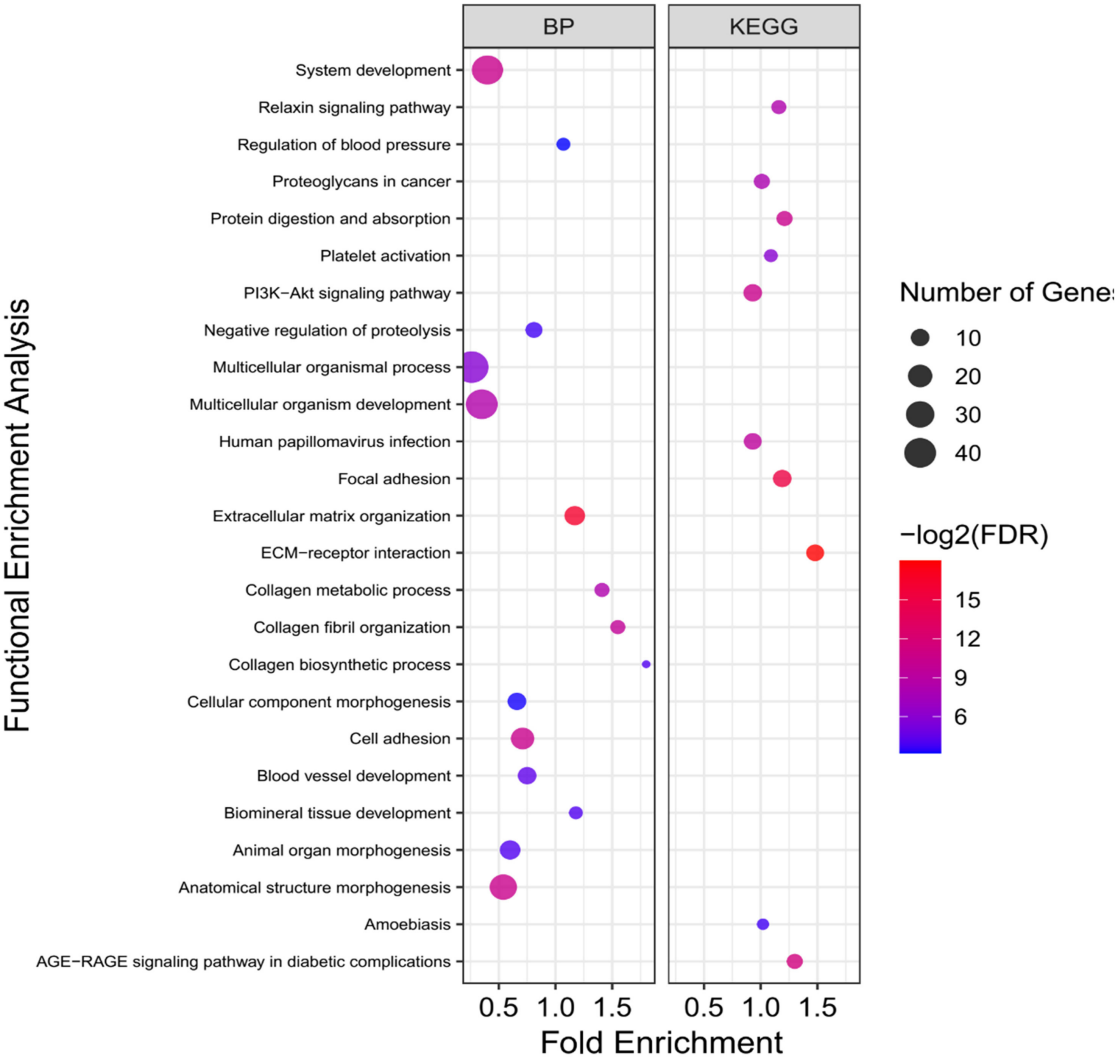
Functional enrichment analysis results of pink sub-network. Size and color of points represent -Log2 of FDR and number of genes associated with each term, respectively. BP, Biological processes.

The resulting PPIs networks were subjected to module analysis with *k* means clustering approach and three significant sub-networks were identified in each network. In this regard, three sub-networks including 22 nodes and 20 edges in sub-network orange (*p*-value = 6.51E−09), 24 nodes and 55 edges in yellow sub-network (*p*-value = 5.49E−11) and 20 nodes and 16 edges in light-blue sub-network (*p*-value = 2.89E−06) were found in up-regulated DEGs (Figure 4). Functional enrichment analysis of the clusters showed only six significant biological processes in orange sub-network including “gene expression,” “RNA metabolic process,” and “translation.” On the other hand, 34 nodes and 41 edges in green sub-network (*p*-value < 1.0e−16), 64 nodes and 90 edges in pink sub-network (*p*-value < 1.0e−16) and 51 nodes and 105 edges in purple sub-network (*p*-value < 1.0e−16) were found in the down-regulated DEGs (Figure 5). Genes of green sub-network were significantly enriched in “Lipid biosynthetic process” biological process (FDR < 0.04) and “Biosynthesis of unsaturated fatty acids” KEGG pathway (FDR < 0.01). Moreover, 17 biological processes (such as “Extracellular matrix organization” and “Anatomical structure morphogenesis”) and 11 KEEG (such as “ECM-receptor interaction,” “PI3K-Akt signaling pathway,” and “Focal adhesion”) were identified in pink sub-network (Figure 6). No significant term was found in purple sub-network.

## Discussion

To date, several transcriptome-based studies have been conducted to identify DEGs related to fat-tail formation through comparison of fat- and thin-tailed sheep breeds (Table 1). However, one of the main problems of these studies is a small number of biological replicates due to the cost of sequencing, which can lead to decrease the statistical power to detect DEGs. In this regard, the variation among the identified DEGs by different datasets (Figure 2) emphasized the importance of meta-analysis to obtain core genes that are consistently DEGs across multiple studies. Some of the lack of agreement from one study to another can be attributed to differences in the experimental conditions such as environmental variables, bioinformatics pipeline, etc.

It should be noted that various sheep breeds have been used in the six studies, however these breeds can be used to identify core genes involved in fat deposition in the fat-tail of sheep. While vertebrate species differ in many phenotypic traits, however, organ physiology in mammals are conserved. Furthermore, it is well-known that many biological processes and pathways are deeply conserved among the species (36). Based on this idea that many developmental gene expression patterns are conserved across mammals, rodents can be applied as models of human tissues physiology (37). Previous studies in this area have shown that inter-study distances between similar tissues of various species were generally less than intra-study distances among various tissues, implying that meta-analysis of using RNA-Seq data from different breeds can provide insights into the molecular mechanisms behind the mammalian tissues (38). Here, a meta-analysis was performed to find the common core DEGs across six studies and remove the inconsistencies in these datasets to obtain a deeper biological insight associated with fat deposition in tail of sheep, compared to that achieved through single dataset. These core genes can be considered as putative candidates involved in fat-tail development regardless of the different characteristics of the studies. Therefore, our aim was to shed light into the molecular mechanisms conserved across the sheep breeds instead of identifying specific mechanisms in each breed.

To better integrate the datasets, a standard and similar bioinformatics workflow was applied to analyze the raw RNA-Seq data from the studies, in which different pipelines were used. To further evaluate the consistency of the DEGs across the datasets, Jackknife sensitivity analysis was applied and revealed that most of the DEGs (>80%) passed at least 50% of the Jackknife tests, which indicates the robustness of the results and independency of these genes to a single study. It is worth to note that in this study only DEGs with the same pattern of expression over half of the datasets were considered (500 DEGs including 221 up- and 279 down-regulated genes in fat- against thin-tailed sheep breeds), which make them more robust and constitutes a suitable way for better understanding the regulatory mechanisms of fat-tail development. To assess potential functions of DEGs in fat-tail development, we investigate their relationships with the QTLs related to sheep fatness and found that they were significantly enriched in these QTLs. These findings reinforced that the identified DEGs may play important roles in fat-tail development in sheep.

GO terms as well as KEGG pathways associated with fat metabolism were the most enriched terms in the DEGs, which explains the fact that these genes can be related to fat-tail formation (Supplementary material 3). Some of the DEGs grouped under terms that were directly or indirectly related to fat metabolism, including *LEPRRBP4, CSNK2B, EPHX1, SCD, ALOX15, SCD5, HSPA6, ACLY, ELOVL6, FOXO3, PPP5C, DAGLA, ACSF2, DHCR24, JUNB, FNDC5, FMO2, FOS, FRZB, ZBTB16, LSS, COL5A1, SLC27A2, SPTLC3, PLPP3, ZBTB20, SMPDL3B, LBP, LPIN1, IL6, PDGFRA, CCL21, SPP1, PAI-1, HSD11B1, MTMR4, S100A1, COL6A3, TNNC2, ITGA1, SCG5, NR4A1, DUSP1*, and *APOE* (6, 8–14, 17, 39–41). Most of these genes passed at least 50% of the Jackknife tests or reside in the QTLs previously found to be linked to sheep fatness (Supplementary material 2, 4). Some of these genes require particular attention considering that they have been previously linked to fat deposition.

PPIs network construction by STRING database confirmed that up- and down-regulated DEGs were members of functional interaction networks. It is well-known that genes from the same sub-network in a PPI network more probably play similar roles and are implicated in the same biological functions. To investigate this hypothesis, sub-network analysis on the PPI network results was performed and two of the three identified sub-networks in the down-regulated DEGs were significantly enriched in different GO terms and KEGG pathways. Hence, it might be possible to hypothesize that genes of green sub-network may be involved in fat deposition in tail of sheep breeds, as “Biosynthesis of unsaturated fatty acids” and “Lipid biosynthetic process” were significantly enriched (Figure 5). Some genes of this sub-network that may be of particular interests are *SCD, SCD5, ELOVL6, ACLY, DHCR24, SPTLC3, LSS, LPIN1, SLC27A2, CKAP2, FOS, JUNB, FOXO3, EBF2*, and *MYBL2*, which are located in the fatness-related QTLs regions or passed at least 50% of the Jackknife tests (Supplementary material 2, 4). In our previous study we hypothesized that down-regulation of the related genes to lipid metabolism in fa-tailed breed may be associated with other pathways than fat deposition such as fat composition (6) or fatty acid oxidation. It has been demonstrated that the breed has significant effect on fatty acid composition of tail fat (42). Here, the similar results were obtained as some members of green sub-network were involved in the pathways associated with fat composition or fatty acid oxidation such as *SCD, SCD5, ELOVL6, ACLY, SLC27A2*, and *LPIN1*.

Isoforms of stearoyl CoA desaturase (*SCD* and *SCD5*) play important roles in desaturation of saturated fatty acids. Lower expression of these genes has been previously reported in fat-tailed than thin-tailed sheep breeds (11, 13), which can cause lower content of saturated fatty acids in fat-tailed breeds. In chicken, *SCD5* expression was significantly correlated with levels of stearidonic acid (43), which might lead to suppress adipocyte differentiation (44). Very long-chain fatty acids protein 6 (*ELOVL6*) is well-known as a major regulator of fatty acid composition in mammals (45). The first step of *de novo* lipogenesis, the conversion of citrate into acetyl-CoA, is catalyzed by *ACLY*. This product, acetyl-CoA, can then be used as building block for long chain fatty acids, cholesterol synthesis and/or histone acetylation (46). It has been shown that *ACLY*-deficient adipocytes accumulate lipid *in vivo* and display some differences in fatty acid content and synthesis (47). Mammals express three lipin genes (*LPIN1-3*) that their functions are evolutionarily conserved. One of the important roles of these genes is transcriptional co-regulators of gene expression in the nucleus to promote fatty acid oxidation (48). In accordance with our results, it was reported that the role of LPIN1 might be closely associated with fatty acid oxidation in the bovine liver (49). Solute carrier family 27 member 2 (*SLC27A2*) is a transmembrane transporter coenzyme that participates in the long-chain fatty acid beta-oxidation (50) as well as plays a key role in fatty acid degradation (51). *SLC27A2* was identified to be closely associated with tail phenotype in Zhang et al. study that investigated the transcriptome profiles of fat deposition in tail of sheep (17). A recent study investigated the correlation of slow-growing meat type chicken liver gene expression with abdominal fat deposition using a time-course transcriptomic study (from the embryonic to the egg-producing period) and reported *CKAP2* as one of the important candidates involved in fat metabolism (52). Furthermore, in green sub-network, five genes including *JUNB, FOXO3, EBF2, FOS*, and *MYBL2* were transcription factors with known roles in lipid metabolism, which can be considered as regulators of this sub-network. A genome-wide association study showed that *MYBL2* may be involved in abdominal fat deposition in chickens (53). *JUNB* is a transcription factor whose role in lipid metabolism and fat cell differentiation has been documented (54). Important roles of *FOXO* protein family in energy homeostasis and lipid metabolism have been highlighted in previous studies (55). Based on our findings, lower expression of members of the green sub-network in fat-tailed sheep breeds are reasonable and might be potential candidate contributing to shaping fat-tail phenotype through regulating fat composition or fatty acid oxidation.

On the other hand, some pathways related to the fat metabolism including “Extracellular matrix (ECM)-receptor interaction,” “Focal adhesion,” and “PI3K-Akt signaling pathway” were significantly enriched in the pink sub-network, which can further contribute to understanding the fat-tail development in sheep (Figure 6). All the genes associated with these pathways (*SPP1, COL1A1, ITGA2, ITGA1, VEGFA, COL6A6, COL4A6, PDGFRA, COL6A3*, and *SDC1*) were located in the QTLs related to fatness or passed at least 50% of the Jackknife tests (Supplementary material 2, 4), which indicated their potential involvement in fat-tail morphogenesis. Focal adhesion is related to ECM and act as mechanical linkages to the extracellular matrix (56). Hence, the DEGs belonging to these two pathways were very similar (Supplementary material 3). ECM is essential for tissue homeostasis and consists of a complex mixture of functional macromolecules in adipose tissue, such as glycosaminoglycans, collagen, elastin, fibronectin, and lammin (57). The ECM communicates with cells through cell surface-related elements such as integrin and regulates cell activities such as differentiation, proliferation, migration, adhesion and apoptosis (58). ECM receptor interaction signaling pathway has been reported as a significant enriched pathway in the DEGs between fat- and thin-tailed sheep breeds in several previous studies (6, 11, 16) as well as in the DEGs between lean and obese human (59). Moreover, interaction between ECM components and transmembrane receptors of fat cells have been demonstrated to be associated with depot-specific adipogenesis in bovine (60). However, we believed that research studies in animal filed have paid no sufficient attention to investigate the role of the ECM receptor interaction pathway in fat metabolism as well as fat-tail formation in sheep. ECM was detected as an inhibited KEGG pathway during differentiation of human mesenchymal stromal-cells into adipocytes. Down-regulated genes belonging to this pathway were in agreement with our results like collagen subunits IV, V and VI (*COL1A1, COL6A6, COL4A6*, and *COL6A3*), different integrins 1 and 2 (*ITGA1* and *ITGA2*) and proteoglycans like syndecan 1 and 4 (*SDC1*). Down-regulation of these genes are attributed to cytoskeleton reorganization during adipogenesis (61). Collagen type IV denaturation is reported to be associated with adipogenic differentiation (62). In a comprehensive assessment to study the role of *COL6A3* in human obesity and diabetes, it was revealed that *COL6A3* expression increased after weight loss and showed a negative correlation with obesity, which is in good agreement with our findings in the current study (63). Moreover, it has been shown that integrin α5 is down-regulated during adipogenesis in 3T3-L1 cells. Therefore, up regulation of this gene inhibits cellular differentiation (64). Accordingly, our findings let hypothesize that ECM might mediates a mechanism involved in the differentiation of fat-tail adipocytes and lipid metabolism, thereby changing the fat-tail morphology of various sheep breeds.

Phosphatidylinositol 3-kinase/protein kinase B (PI3K/AKT) signaling pathway, as the other enriched pathway in pink sub-network, is a key pathway that specifically phosphorylates phosphatidylinositol, generate an intracellular second messengers and mediates glucose and lipid metabolism (65). It is reported that PI3K/AKT signaling pathway has different functions in various adipocytes, as promote and inhibit the proliferation/differentiation in human adipocytes and 3T3-L1, respectively (66, 67). It has been shown that inhibition of this pathway in children with simple obesity participates in the occurrence and progression of obesity (68). In a recent study, PI3K/AKT signaling pathways was enriched in the target genes of differentially expressed miRNAs between fat- and thin-tailed sheep breeds (69) and suggested to be involved in biological processes related to fat deposition in fat-tail tissue. In the current study, enrichment of this pathway in the down-regulated genes in fat-tailed breeds suggesting that it may inhibits the proliferation and differentiation of lipid metabolism in fat-tail tissue and leads to differences in fat deposition between the different sheep breeds. Some of the DEGs belonging to PI3K/AKT signaling pathway in this study were included *SPP1, PDGFRA, VEGFA*, and *TNC*. Positive and negative effects of *SPP1* in fat deposition had been demonstrated in previous reports. Studies have established that this gene is synthesized by adipocytes and its higher expression is related to fat deposition (70). In contrast, it is reported that interaction of *SPP1* with integrin αv/β1 inhibits the adipogenesis of mesenchymal stem cells (71). In agreement with our results, a recent study found that *SPP1* negatively regulated adipogenic differentiation of peripheral blood-derived mesenchymal stem cells and interaction between novel-miR-659 and *SPP1* coregulate fat deposition in castrated male pigs (40). All these findings support the potential function of PI3K/AKT signaling pathway and its related genes in mediating lipolysis and energy expenditure, which can be led to lower fat deposition.

Some of the up-regulated DEGs in this study have been previously reported as candidate genes involved in fat-tail/fatness development or as DEG associated with fat metabolism in animal. Moreover, they were found in the fatness-related QTLs or passed at least 50% of the Jackknife tests (Supplementary material 2, 4). Some of the most important genes in light blue sub-network (Figure 4) were *IL6, RBP4, LEPR, PAI*-1, *CSNK2B*, and *MTMR4*. Recent studies highlighted the role of interleukins in lipid metabolism (49). Interleukin-6 (IL-6) is known as a key regulator of adipose homeostasis in obesity. It is worth to note that in Farhadi et al., study this gene was down-regulated in fat-tailed breed and they suggested it as a potential candidate gene in fat-tail formation. However, our analysis revealed that this gene was up-regulated in fat-tailed breed in all studies, except Farhadi et al., study. In this regard, results of a study showed that expression of *IL-6* in lymphedema [a morbid disease characterized by adipose deposition (72)] is associated with adipose deposition. Since, *IL-6* was identified as a highly connected gene in light blue sub-network (Figure 4), the other genes in this cluster are expected to be potential candidate genes in fat-tail formation. Interestingly, most of the connected genes with *IL-6* were reported to be involved in fat deposition processes, which reinforce the importance of light blue sub-network in fat deposition. For example, retinol binding protein 4 (*RBP4*), which is a novel adipokine, is mainly secreted by adipocytes and is related to obesity. This gene contributes to systemic insulin resistance that can lead to fat deposition (73). Leptin receptor (*LEPR*), as other gene in light blue sub-network that is also located in fatness-related QTLs regions, was reported as candidate gene affecting fat deposition in pig (74). Higher levels of plasminogen activator inhibitor-1 (*PAI-1*) in plasma was reported to be a biochemical marker of obesity (75). The association between *PAI-1* and fat deposition had been well-established in animal studies and the location of this gene in the QTLs linked to sheep fatness make it interesting for further functional investigations (76).

*EPHX1, HSD11B1, FMO2, S100A1*, and *HSPA6* were some of the important up-regulated genes in orange sub-network (Figure 4). Association between *EPHX1* and adipogenesis in mesenchymal stem cells through the activation of cryoprotective lipid mediators had been explained previously (77). Rosu-Myles et al., reported that *EPHX1* expressing cells in human stromal cultures can be led to increased numbers of cells that have committed to the adipocyte lineage (78). *FMO2* is a member of the *FMO* gene family and catalyze the *NADPH*-dependent oxidative metabolism of a wide array of foreign chemicals as well as is involved in fat deposition, adipogenesis and fatty acid biosynthesis (12). *FMO3*, as other member of this family, was reported as important candidate in fat metabolism of sheep through inhibiting fatty acid oxidation (14). Consistent with these observations, *FMOs* 1, 2, and 4 knockout mice exhibited a lean phenotype and stored less triglycerides in their white adipose tissue compared to wild-type mice, despite similar food intake (79). *HSD11B1* is well-known to be closely related to the accumulation of abdominal fat (80). Recently, an RNA-Seq study was performed to explore the effects of castration on fat deposition in different parts of pigs and *HSD11B1* was reported as a factor affects glucose uptake by adipocytes and leads to obesity (81). *HSPA6* was reported as an important regulator of fatty acid metabolism in the skeletal muscles of sheep (39). Adipogenesis regulatory factor (*ADIRF*), as other important up-regulated gene, promotes adipocyte differentiation by enhancing the expression of peroxisome proliferator-activated receptor gamma (*PPARG*) and CCAAT-enhancer-binding protein alpha (*CEBPA*) in 3T3 L1 cells and play an important role in fat cell development. Higher expression of this gene in obese individuals, suggesting a role for *ADIRF* in the development of obesity (82). Angiopoietin-like 8 (*ANGPTL8*) exhibits its effects by inducing insulin receptor to inhibits lipolysis and controls post-prandial fat storage in white adipose tissue. This gene directs fatty acids to adipose tissue for storage during the fed state. Serpin family E member 1 (*SERPINE1*) was highly expressed in obese individuals and demonstrated as a key gene associated with the network pathway analysis of obesity (83). Altogether, these results support the potential role of up-regulated genes, especially light blue and orange sub-networks in fat deposition in tail of sheep.

Totally, 170 unique DEGs (77 up- and 93 down-regulated) were found in meta-analysis that were not identified as DEGs in individual studies (Figure 2), which can be attributed to the more statistical power of meta-analysis than individual studies for identifying new candidate genes associated with fat-tail formation. Of which, 38 DEGs (17 up and 21 down-regulated) were located in QTLs regions related to fatness of sheep, which further support that their functions might be relevant. Some of these genes have been previously reported to be related to lipid metabolism including *NR4A1, ACSF2, MYC, SPP1, PLPP3, PHOSPHO1*, and *ACP6*. For example, *NR4A1* encodes a nuclear receptor (transcription factor) that is involved in regulation of lipid metabolism and modulating lipolysis in muscle (41). It is reported that female *NR4A1* deficient mice exhibited higher fat mass compared to wild-type mice, under the same high-fat diet (84). This gene plays a vital role in the regulation of liver fat content (41). Expression of *NR4A1* is reported to be negatively correlated with body-fat content and insulin sensitivity, as its expression was significantly lower in the muscle of obese men in comparison to lean men (85). *MYC*, which is a transcription factor, plays vital roles in lipid metabolism (86). Furthermore, *ACSF2* is involved in the acyl-CoA metabolic process and malonyl-CoA metabolic process in mammals as well as reported to be associated with avian lipid metabolism (87, 88). These findings suggested a link between differentially expressed of these genes and fat-tail development in sheep.

## Conclusion

In this study, a meta-analysis of six RNA-Seq datasets that compared transcriptome profiles of fat- and thin-tailed sheep breeds, were performed. By considering the expression pattern of DEGs across the different studies and performing Jackknife analysis, a list of robust DEGs were obtained, which were enriched in the QTLs related to sheep fatness. Moreover, functional interactions among the DEGs were confirmed using PPIs network analysis. Functional enrichment analysis showed that the DEGs were enriched in the GO terms/KEGG pathways related to fat metabolism such as “fatty-acyl-CoA biosynthetic process,” “ECM-receptor interaction,” and “PI3K-Akt signaling pathway.” Cluster analysis of the PPIs network led to identification several sub-networks that were directly or indirectly involved in fat deposition. Some down-regulated DEGs in green and pink sub-networks were *SCD, SCD5, ELOVL6, ACLY, SLC27A2, LPIN1, COL1A1, COL6A6, COL4A6, COL6A3, ITGA1, ITGA2, SDC1*, and *SPP1*, which probably promote the development of fat-tail through regulating lipolysis or fatty acid oxidation. In contrast, up-regulated DEGs such as *IL6, RBP4, LEPR, PAI-1, CSNK2B, MTMR4, EPHX1, HSD11B1, FMO2, S100A1*, and *HSPA6* were mainly enriched in green and pink sub-networks and may contribute to a network controlling fat accumulation in tail of sheep breed through mediating adipogenesis and fatty acid biosynthesis. Overall, our meta-analysis successfully identified a core set of DEGs associated with lipid metabolism including well-known genes related to fat deposition as well as newly identified genes such as *NR4A1* and *ACSF2*. Therefore, it is reasonable to infer that the suggested sub-networks and their gene members might be potential candidate contributing to shaping fat-tail phenotype. Although our findings were in good agreement with previous studies, however they are in transcriptome level and follow-up functional studies are required to investigate the mechanisms by which these genes contribute to the fat deposition in tail of sheep breeds.

## Data availability statement

Publicly available datasets were analyzed in this study. The names of the repository/repositories and accession number(s) can be found in the article/Supplementary material.

## Author contributions

MB conceived the ideas. MB and SH designed study and analyzed the data. MB, SH, and AS interpreted the data and wrote the main manuscript text. All authors read and approved the final manuscript.
