# Supplementary material for: Meta-analysis of RNA-Seq datasets highlights novel genes/pathways involved in fat deposition in fat-tail of sheep
Source: Front Vet Sci. 2023 May 12;10:1159921. doi: 10.3389/fvets.2023.1159921 (PMC10213422; doi:10.3389/fvets.2023.1159921)
Supplement: Supplementary file 5 [file Table_5.DOCX]

**Supplementary File S5. The used R code to analyze the RNA-Seq datasets.**

setwd("H:/6-My Students/MSc/20-Hosseini/2-Analysis")

library("DESeq2")

library("IHW")

library("ggplot2")

################################################################# DESeq2

#################################################### 1_Lori-Zel

########################## Combining the count files

cntdir_1_Lori_Zel <- "Htseq_Results/1_Lori-Zel"

pat_1_Lori_Zel <- ".count"

myfiles_1_Lori_Zel <- list.files(path = cntdir_1_Lori_Zel,

pattern = pat_1_Lori_Zel,

all.files = TRUE,

recursive = FALSE,

ignore.case = FALSE,

include.dirs = FALSE)

DT_1_Lori_Zel <- list()

# read each file as array element of DT and rename the last 2 cols

# we created a list of single sample tables

for (i in 1:length(myfiles_1_Lori_Zel) ) {

infile = paste(cntdir_1_Lori_Zel, myfiles_1_Lori_Zel[i], sep = "/")

DT_1_Lori_Zel[[myfiles_1_Lori_Zel[i]]] <- read.table(infile, header = F, stringsAsFactors = FALSE)

cnts <- gsub("(.*).count", "\\1", myfiles_1_Lori_Zel[i])

colnames(DT_1_Lori_Zel[[myfiles_1_Lori_Zel[i]]]) <- c("ID", cnts)

}

# merge all elements based on first ID columns

data_1_Lori_Zel <- DT_1_Lori_Zel[[myfiles_1_Lori_Zel[1]]]

for (i in 2:length(myfiles_1_Lori_Zel)) {

y_1_Lori_Zel <- DT_1_Lori_Zel[[myfiles_1_Lori_Zel[i]]]

z_1_Lori_Zel <- merge(data_1_Lori_Zel, y_1_Lori_Zel, by = c("ID"))

data_1_Lori_Zel <- z_1_Lori_Zel

}

# ID column becomes rownames

rownames(data_1_Lori_Zel) <- data_1_Lori_Zel$ID

data_1_Lori_Zel <- data_1_Lori_Zel[,-1]

# write summary to file

write.csv(data_1_Lori_Zel, file = "1_Lori_Zel.csv")

########################## End of combining the count files (if you need)

########################## DESeq2

samplenames_1_Lori_Zel=colnames(data_1_Lori_Zel)

condition=c("Fat","Fat","Fat",

"Thin","Thin","Thin")

coldata_1_Lori_Zel=as.data.frame(cbind(samplenames_1_Lori_Zel,condition))

coldata_1_Lori_Zel$condition=factor(coldata_1_Lori_Zel$condition)

coldata_1_Lori_Zel$samplenames_1_Lori_Zel=factor(coldata_1_Lori_Zel$samplenames_1_Lori_Zel)

dds_1_Lori_Zel = DESeqDataSetFromMatrix(countData = data_1_Lori_Zel,

colData = coldata_1_Lori_Zel,

design= ~ condition)

# make 3D PCA plot

vsd_1_Lori_Zel <- vst(dds_1_Lori_Zel, blind=FALSE)

plotPCA(vsd_1_Lori_Zel, intgroup=c("condition")) + geom_text(aes(label=samplenames_1_Lori_Zel),vjust=2)

# DESeq

keep_1_Lori_Zel <- rowSums(counts(dds_1_Lori_Zel)) >= 10

dds_1_Lori_Zel <- dds_1_Lori_Zel[keep_1_Lori_Zel,]

dds_1_Lori_Zel=DESeq(dds_1_Lori_Zel)

resIHW_1_Lori_Zel <- results(dds_1_Lori_Zel, filterFun=ihw)

write.csv(resIHW_1_Lori_Zel,"1_Lori_Zel_DESeq2.csv", row.names=TRUE)

Lori_Zel_DEGs=read.csv("DESeq2_Results/1_Lori_Zel_DESeq2.csv")

Selection=read.csv("DESeq2_Results/1_Lori_Zel_DESeq2.csv")$padj<0.05

Lori_Zel_DEGs=Lori_Zel_DEGs$Selection

Lori_Zel_DEGs <- subset(read.csv("DESeq2_Results/1_Lori_Zel_DESeq2.csv"), padj<0.05, select=c(X))

########################## End of DESeq2

#################################################### End of 1_Lori-Zel

#################################################### 2_HAN_Fat-Thin

########################## Combining the count files

cntdir_2_HAN_Fat_Thin <- "Htseq_Results/2_HAN_Fat-Thin"

pat_2_HAN_Fat_Thin <- ".count"

myfiles_2_HAN_Fat_Thin <- list.files(path = cntdir_2_HAN_Fat_Thin,

pattern = pat_2_HAN_Fat_Thin,

all.files = TRUE,

recursive = FALSE,

ignore.case = FALSE,

include.dirs = FALSE)

DT_2_HAN_Fat_Thin <- list()

# read each file as array element of DT and rename the last 2 cols

# we created a list of single sample tables

for (i in 1:length(myfiles_2_HAN_Fat_Thin) ) {

infile = paste(cntdir_2_HAN_Fat_Thin, myfiles_2_HAN_Fat_Thin[i], sep = "/")

DT_2_HAN_Fat_Thin[[myfiles_2_HAN_Fat_Thin[i]]] <- read.table(infile, header = F, stringsAsFactors = FALSE)

cnts <- gsub("(.*).count", "\\1", myfiles_2_HAN_Fat_Thin[i])

colnames(DT_2_HAN_Fat_Thin[[myfiles_2_HAN_Fat_Thin[i]]]) <- c("ID", cnts)

}

# merge all elements based on first ID columns

data_2_HAN_Fat_Thin <- DT_2_HAN_Fat_Thin[[myfiles_2_HAN_Fat_Thin[1]]]

for (i in 2:length(myfiles_2_HAN_Fat_Thin)) {

y_2_HAN_Fat_Thin <- DT_2_HAN_Fat_Thin[[myfiles_2_HAN_Fat_Thin[i]]]

z_2_HAN_Fat_Thin <- merge(data_2_HAN_Fat_Thin, y_2_HAN_Fat_Thin, by = c("ID"))

data_2_HAN_Fat_Thin <- z_2_HAN_Fat_Thin

}

# ID column becomes rownames

rownames(data_2_HAN_Fat_Thin) <- data_2_HAN_Fat_Thin$ID

data_2_HAN_Fat_Thin <- data_2_HAN_Fat_Thin[,-1]

# write summary to file

write.csv(data_2_HAN_Fat_Thin, file = "2_HAN_Fat_Thin.csv")

########################## End of combining the count files (if you need)

########################## DESeq2

samplenames_2_HAN_Fat_Thin=colnames(data_2_HAN_Fat_Thin)

condition=c("Fat","Fat","Fat",

"Thin","Thin","Thin")

coldata_2_HAN_Fat_Thin=as.data.frame(cbind(samplenames_2_HAN_Fat_Thin,condition))

coldata_2_HAN_Fat_Thin$condition=factor(coldata_2_HAN_Fat_Thin$condition)

coldata_2_HAN_Fat_Thin$samplenames_2_HAN_Fat_Thin=factor(coldata_2_HAN_Fat_Thin$samplenames_2_HAN_Fat_Thin)

dds_2_HAN_Fat_Thin = DESeqDataSetFromMatrix(countData = data_2_HAN_Fat_Thin,

colData = coldata_2_HAN_Fat_Thin,

design= ~ condition)

# make 3D PCA plot

vsd_2_HAN_Fat_Thin <- vst(dds_2_HAN_Fat_Thin, blind=FALSE)

plotPCA(vsd_2_HAN_Fat_Thin, intgroup=c("condition")) + geom_text(aes(label=samplenames_2_HAN_Fat_Thin),vjust=2)

# DESeq

keep_2_HAN_Fat_Thin <- rowSums(counts(dds_2_HAN_Fat_Thin)) >= 10

dds_2_HAN_Fat_Thin <- dds_2_HAN_Fat_Thin[keep_2_HAN_Fat_Thin,]

dds_2_HAN_Fat_Thin=DESeq(dds_2_HAN_Fat_Thin)

resIHW_2_HAN_Fat_Thin <- results(dds_2_HAN_Fat_Thin, filterFun=ihw)

write.csv(resIHW_2_HAN_Fat_Thin,"2_HAN_Fat_Thin_DESeq2.csv", row.names=TRUE)

HAN_Fat_Thin_DEGs <- subset(read.csv("DESeq2_Results/2_HAN_Fat_Thin_DESeq2.csv"), padj<0.05, select=c(X))

########################## End of DESeq2

#################################################### End of 2_HAN_Fat-Thin

#################################################### 3_Lanzhu-Han

########################## Combining the count files

cntdir_3_Lanzhu_Han <- "Htseq_Results/3_Lanzhu-Han"

pat_3_Lanzhu_Han <- ".count"

myfiles_3_Lanzhu_Han <- list.files(path = cntdir_3_Lanzhu_Han,

pattern = pat_3_Lanzhu_Han,

all.files = TRUE,

recursive = FALSE,

ignore.case = FALSE,

include.dirs = FALSE)

DT_3_Lanzhu_Han <- list()

# read each file as array element of DT and rename the last 2 cols

# we created a list of single sample tables

for (i in 1:length(myfiles_3_Lanzhu_Han) ) {

infile = paste(cntdir_3_Lanzhu_Han, myfiles_3_Lanzhu_Han[i], sep = "/")

DT_3_Lanzhu_Han[[myfiles_3_Lanzhu_Han[i]]] <- read.table(infile, header = F, stringsAsFactors = FALSE)

cnts <- gsub("(.*).count", "\\1", myfiles_3_Lanzhu_Han[i])

colnames(DT_3_Lanzhu_Han[[myfiles_3_Lanzhu_Han[i]]]) <- c("ID", cnts)

}

# merge all elements based on first ID columns

data_3_Lanzhu_Han <- DT_3_Lanzhu_Han[[myfiles_3_Lanzhu_Han[1]]]

for (i in 2:length(myfiles_3_Lanzhu_Han)) {

y_3_Lanzhu_Han <- DT_3_Lanzhu_Han[[myfiles_3_Lanzhu_Han[i]]]

z_3_Lanzhu_Han <- merge(data_3_Lanzhu_Han, y_3_Lanzhu_Han, by = c("ID"))

data_3_Lanzhu_Han <- z_3_Lanzhu_Han

}

# ID column becomes rownames

rownames(data_3_Lanzhu_Han) <- data_3_Lanzhu_Han$ID

data_3_Lanzhu_Han <- data_3_Lanzhu_Han[,-1]

# write summary to file

write.csv(data_3_Lanzhu_Han, file = "3_Lanzhu_Han.csv")

########################## End of combining the count files (if you need)

########################## DESeq2

samplenames_3_Lanzhu_Han=colnames(data_3_Lanzhu_Han)

condition=c("Fat","Fat","Fat",

"Thin","Thin","Thin")

coldata_3_Lanzhu_Han=as.data.frame(cbind(samplenames_3_Lanzhu_Han,condition))

coldata_3_Lanzhu_Han$condition=factor(coldata_3_Lanzhu_Han$condition)

coldata_3_Lanzhu_Han$samplenames_3_Lanzhu_Han=factor(coldata_3_Lanzhu_Han$samplenames_3_Lanzhu_Han)

dds_3_Lanzhu_Han = DESeqDataSetFromMatrix(countData = data_3_Lanzhu_Han,

colData = coldata_3_Lanzhu_Han,

design= ~ condition)

# make 3D PCA plot

vsd_3_Lanzhu_Han <- vst(dds_3_Lanzhu_Han, blind=FALSE)

plotPCA(vsd_3_Lanzhu_Han, intgroup=c("condition")) + geom_text(aes(label=samplenames_3_Lanzhu_Han),vjust=2)

# DESeq

keep_3_Lanzhu_Han <- rowSums(counts(dds_3_Lanzhu_Han)) >= 10

dds_3_Lanzhu_Han <- dds_3_Lanzhu_Han[keep_3_Lanzhu_Han,]

dds_3_Lanzhu_Han=DESeq(dds_3_Lanzhu_Han)

resIHW_3_Lanzhu_Han <- results(dds_3_Lanzhu_Han, filterFun=ihw)

write.csv(resIHW_3_Lanzhu_Han,"3_Lanzhu_Han_DESeq2.csv", row.names=TRUE)

Lanzhu_Han_DEGs <- subset(read.csv("DESeq2_Results/3_Lanzhu_Han_DESeq2.csv"), padj<0.05, select=c(X))

########################## End of DESeq2

#################################################### End of 3_Lanzhu-Han

#################################################### 4_Dorper-Hu

########################## Combining the count files

cntdir_4_Dorper_Hu <- "Htseq_Results/4_Dorper-Hu"

pat_4_Dorper_Hu <- ".count"

myfiles_4_Dorper_Hu <- list.files(path = cntdir_4_Dorper_Hu,

pattern = pat_4_Dorper_Hu,

all.files = TRUE,

recursive = FALSE,

ignore.case = FALSE,

include.dirs = FALSE)

DT_4_Dorper_Hu <- list()

# read each file as array element of DT and rename the last 2 cols

# we created a list of single sample tables

for (i in 1:length(myfiles_4_Dorper_Hu) ) {

infile = paste(cntdir_4_Dorper_Hu, myfiles_4_Dorper_Hu[i], sep = "/")

DT_4_Dorper_Hu[[myfiles_4_Dorper_Hu[i]]] <- read.table(infile, header = F, stringsAsFactors = FALSE)

cnts <- gsub("(.*).count", "\\1", myfiles_4_Dorper_Hu[i])

colnames(DT_4_Dorper_Hu[[myfiles_4_Dorper_Hu[i]]]) <- c("ID", cnts)

}

# merge all elements based on first ID columns

data_4_Dorper_Hu <- DT_4_Dorper_Hu[[myfiles_4_Dorper_Hu[1]]]

for (i in 2:length(myfiles_4_Dorper_Hu)) {

y_4_Dorper_Hu <- DT_4_Dorper_Hu[[myfiles_4_Dorper_Hu[i]]]

z_4_Dorper_Hu <- merge(data_4_Dorper_Hu, y_4_Dorper_Hu, by = c("ID"))

data_4_Dorper_Hu <- z_4_Dorper_Hu

}

# ID column becomes rownames

rownames(data_4_Dorper_Hu) <- data_4_Dorper_Hu$ID

data_4_Dorper_Hu <- data_4_Dorper_Hu[,-1]

# write summary to file

write.csv(data_4_Dorper_Hu, file = "4_Dorper_Hu.csv")

########################## End of combining the count files (if you need)

########################## DESeq2

samplenames_4_Dorper_Hu=colnames(data_4_Dorper_Hu)

condition=c("Fat","Fat","Fat",

"Thin","Thin","Thin")

coldata_4_Dorper_Hu=as.data.frame(cbind(samplenames_4_Dorper_Hu,condition))

coldata_4_Dorper_Hu$condition=factor(coldata_4_Dorper_Hu$condition)

coldata_4_Dorper_Hu$samplenames_4_Dorper_Hu=factor(coldata_4_Dorper_Hu$samplenames_4_Dorper_Hu)

dds_4_Dorper_Hu = DESeqDataSetFromMatrix(countData = data_4_Dorper_Hu,

colData = coldata_4_Dorper_Hu,

design= ~ condition)

# make 3D PCA plot

vsd_4_Dorper_Hu <- vst(dds_4_Dorper_Hu, blind=FALSE)

plotPCA(vsd_4_Dorper_Hu, intgroup=c("condition")) + geom_text(aes(label=samplenames_4_Dorper_Hu),vjust=2)

# DESeq

keep_4_Dorper_Hu <- rowSums(counts(dds_4_Dorper_Hu)) >= 10

dds_4_Dorper_Hu <- dds_4_Dorper_Hu[keep_4_Dorper_Hu,]

dds_4_Dorper_Hu=DESeq(dds_4_Dorper_Hu)

resIHW_4_Dorper_Hu <- results(dds_4_Dorper_Hu, filterFun=ihw)

write.csv(resIHW_4_Dorper_Hu,"4_Dorper_Hu_DESeq2.csv", row.names=TRUE)

Dorper_Hu_DEGs <- subset(read.csv("DESeq2_Results/4_Dorper_Hu_DESeq2.csv"), padj<0.05, select=c(X))

########################## End of DESeq2

#################################################### End of 4_Dorper-Hu

#################################################### 5_Hulun_Fat_Thin

########################## Combining the count files

cntdir_5_Hulun_Fat_Thin <- "Htseq_Results/5_Hulun_Fat_Thin"

pat_5_Hulun_Fat_Thin <- ".count"

myfiles_5_Hulun_Fat_Thin <- list.files(path = cntdir_5_Hulun_Fat_Thin,

pattern = pat_5_Hulun_Fat_Thin,

all.files = TRUE,

recursive = FALSE,

ignore.case = FALSE,

include.dirs = FALSE)

DT_5_Hulun_Fat_Thin <- list()

# read each file as array element of DT and rename the last 2 cols

# we created a list of single sample tables

for (i in 1:length(myfiles_5_Hulun_Fat_Thin) ) {

infile = paste(cntdir_5_Hulun_Fat_Thin, myfiles_5_Hulun_Fat_Thin[i], sep = "/")

DT_5_Hulun_Fat_Thin[[myfiles_5_Hulun_Fat_Thin[i]]] <- read.table(infile, header = F, stringsAsFactors = FALSE)

cnts <- gsub("(.*).count", "\\1", myfiles_5_Hulun_Fat_Thin[i])

colnames(DT_5_Hulun_Fat_Thin[[myfiles_5_Hulun_Fat_Thin[i]]]) <- c("ID", cnts)

}

# merge all elements based on first ID columns

data_5_Hulun_Fat_Thin <- DT_5_Hulun_Fat_Thin[[myfiles_5_Hulun_Fat_Thin[1]]]

for (i in 2:length(myfiles_5_Hulun_Fat_Thin)) {

y_5_Hulun_Fat_Thin <- DT_5_Hulun_Fat_Thin[[myfiles_5_Hulun_Fat_Thin[i]]]

z_5_Hulun_Fat_Thin <- merge(data_5_Hulun_Fat_Thin, y_5_Hulun_Fat_Thin, by = c("ID"))

data_5_Hulun_Fat_Thin <- z_5_Hulun_Fat_Thin

}

# ID column becomes rownames

rownames(data_5_Hulun_Fat_Thin) <- data_5_Hulun_Fat_Thin$ID

data_5_Hulun_Fat_Thin <- data_5_Hulun_Fat_Thin[,-1]

# write summary to file

write.csv(data_5_Hulun_Fat_Thin, file = "5_Hulun_Fat_Thin.csv")

########################## End of combining the count files (if you need)

########################## DESeq2

samplenames_5_Hulun_Fat_Thin=colnames(data_5_Hulun_Fat_Thin)

condition=c("Fat","Fat","Fat",

"Thin","Thin","Thin")

coldata_5_Hulun_Fat_Thin=as.data.frame(cbind(samplenames_5_Hulun_Fat_Thin,condition))

coldata_5_Hulun_Fat_Thin$condition=factor(coldata_5_Hulun_Fat_Thin$condition)

coldata_5_Hulun_Fat_Thin$samplenames_5_Hulun_Fat_Thin=factor(coldata_5_Hulun_Fat_Thin$samplenames_5_Hulun_Fat_Thin)

dds_5_Hulun_Fat_Thin = DESeqDataSetFromMatrix(countData = data_5_Hulun_Fat_Thin,

colData = coldata_5_Hulun_Fat_Thin,

design= ~ condition)

# make 3D PCA plot

vsd_5_Hulun_Fat_Thin <- vst(dds_5_Hulun_Fat_Thin, blind=FALSE)

plotPCA(vsd_5_Hulun_Fat_Thin, intgroup=c("condition")) + geom_text(aes(label=samplenames_5_Hulun_Fat_Thin),vjust=2)

# DESeq

keep_5_Hulun_Fat_Thin <- rowSums(counts(dds_5_Hulun_Fat_Thin)) >= 10

dds_5_Hulun_Fat_Thin <- dds_5_Hulun_Fat_Thin[keep_5_Hulun_Fat_Thin,]

dds_5_Hulun_Fat_Thin=DESeq(dds_5_Hulun_Fat_Thin)

resIHW_5_Hulun_Fat_Thin <- results(dds_5_Hulun_Fat_Thin, filterFun=ihw)

write.csv(resIHW_5_Hulun_Fat_Thin,"5_Hulun_Fat_Thin_DESeq2.csv", row.names=TRUE)

Hulun_Fat_Thin_DEGs <- subset(read.csv("DESeq2_Results/5_Hulun_Fat_Thin_DESeq2.csv"), padj<0.05, select=c(X))

########################## End of DESeq2

#################################################### End of 5_Hulun_Fat_Thin

#################################################### 6_Ghezel_Zel

########################## Combining the count files

cntdir_6_Ghezel_Zel <- "Htseq_Results/6_Ghezel_Zel"

pat_6_Ghezel_Zel <- ".count"

myfiles_6_Ghezel_Zel <- list.files(path = cntdir_6_Ghezel_Zel,

pattern = pat_6_Ghezel_Zel,

all.files = TRUE,

recursive = FALSE,

ignore.case = FALSE,

include.dirs = FALSE)

DT_6_Ghezel_Zel <- list()

# read each file as array element of DT and rename the last 2 cols

# we created a list of single sample tables

for (i in 1:length(myfiles_6_Ghezel_Zel) ) {

infile = paste(cntdir_6_Ghezel_Zel, myfiles_6_Ghezel_Zel[i], sep = "/")

DT_6_Ghezel_Zel[[myfiles_6_Ghezel_Zel[i]]] <- read.table(infile, header = F, stringsAsFactors = FALSE)

cnts <- gsub("(.*).count", "\\1", myfiles_6_Ghezel_Zel[i])

colnames(DT_6_Ghezel_Zel[[myfiles_6_Ghezel_Zel[i]]]) <- c("ID", cnts)

}

# merge all elements based on first ID columns

data_6_Ghezel_Zel <- DT_6_Ghezel_Zel[[myfiles_6_Ghezel_Zel[1]]]

for (i in 2:length(myfiles_6_Ghezel_Zel)) {

y_6_Ghezel_Zel <- DT_6_Ghezel_Zel[[myfiles_6_Ghezel_Zel[i]]]

z_6_Ghezel_Zel <- merge(data_6_Ghezel_Zel, y_6_Ghezel_Zel, by = c("ID"))

data_6_Ghezel_Zel <- z_6_Ghezel_Zel

}

# ID column becomes rownames

rownames(data_6_Ghezel_Zel) <- data_6_Ghezel_Zel$ID

data_6_Ghezel_Zel <- data_6_Ghezel_Zel[,-1]

# write summary to file

write.csv(data_6_Ghezel_Zel, file = "6_Ghezel_Zel.csv")

########################## End of combining the count files (if you need)

########################## DESeq2

samplenames_6_Ghezel_Zel=colnames(data_6_Ghezel_Zel)

condition=c("Fat","Fat","Fat",

"Thin","Thin","Thin","Thin")

coldata_6_Ghezel_Zel=as.data.frame(cbind(samplenames_6_Ghezel_Zel,condition))

coldata_6_Ghezel_Zel$condition=factor(coldata_6_Ghezel_Zel$condition)

coldata_6_Ghezel_Zel$samplenames_6_Ghezel_Zel=factor(coldata_6_Ghezel_Zel$samplenames_6_Ghezel_Zel)

dds_6_Ghezel_Zel = DESeqDataSetFromMatrix(countData = data_6_Ghezel_Zel,

colData = coldata_6_Ghezel_Zel,

design= ~ condition)

# make 3D PCA plot

vsd_6_Ghezel_Zel <- vst(dds_6_Ghezel_Zel, blind=FALSE)

plotPCA(vsd_6_Ghezel_Zel, intgroup=c("condition")) + geom_text(aes(label=samplenames_6_Ghezel_Zel),vjust=2)

# DESeq

keep_6_Ghezel_Zel <- rowSums(counts(dds_6_Ghezel_Zel)) >= 10

dds_6_Ghezel_Zel <- dds_6_Ghezel_Zel[keep_6_Ghezel_Zel,]

dds_6_Ghezel_Zel=DESeq(dds_6_Ghezel_Zel)

resIHW_6_Ghezel_Zel <- results(dds_6_Ghezel_Zel, filterFun=ihw)

write.csv(resIHW_6_Ghezel_Zel,"6_Ghezel_Zel_DESeq2.csv", row.names=TRUE)

Ghezel_Zel_DEGs <- subset(read.csv("DESeq2_Results/6_Ghezel_Zel_DESeq2.csv"), padj<0.05, select=c(X))

########################## End of DESeq2

#################################################### End of 6_Ghezel_Zel

################################################################# End of DESeq2

################################################################# metaRNASeq

########## Reading the data

d1=read.csv("DESeq2_Results/1_Lori_Zel_DESeq2.csv", header=T)

d1=cbind.data.frame(d1$X, d1$log2FoldChange, d1$pvalue, d1$padj)

colnames(d1)=c("ID", "log2FoldChange_1", "pvalue_1", "padj_1" )

d2=read.csv("DESeq2_Results/2_HAN_Fat_Thin_DESeq2.csv", header=T)

d2=cbind.data.frame(d2$X, d2$log2FoldChange, d2$pvalue, d2$padj)

colnames(d2)=c("ID", "log2FoldChange_2", "pvalue_2", "padj_2" )

d3=read.csv("DESeq2_Results/3_Lanzhu_Han_DESeq2.csv", header=T)

d3=cbind.data.frame(d3$X, d3$log2FoldChange, d3$pvalue, d3$padj)

colnames(d3)=c("ID", "log2FoldChange_3", "pvalue_3", "padj_3" )

d4=read.csv("DESeq2_Results/4_Dorper_Hu_DESeq2.csv", header=T)

d4=cbind.data.frame(d4$X, d4$log2FoldChange, d4$pvalue, d4$padj)

colnames(d4)=c("ID", "log2FoldChange_4", "pvalue_4", "padj_4" )

d5=read.csv("DESeq2_Results/5_Hulun_Fat_Thin_DESeq2.csv", header=T)

d5=cbind.data.frame(d5$X, d5$log2FoldChange, d5$pvalue, d5$padj)

colnames(d5)=c("ID", "log2FoldChange_5", "pvalue_5", "padj_5" )

d6=read.csv("DESeq2_Results/6_Ghezel_Zel_DESeq2.csv", header=T)

d6=cbind.data.frame(d6$X, d6$log2FoldChange, d6$pvalue, d6$padj)

colnames(d6)=c("ID", "log2FoldChange_6", "pvalue_6", "padj_6" )

########## End of reading the data

########## Merging the data

All= merge(merge(merge(merge(merge(d1, d2, by='ID', all=T), d3, by='ID', all=T),

d4, by='ID', all=T),

d5, by='ID', all=T),

d6, by='ID', all=T)

write.csv(All,"All.csv")

##########

########## Extract different statistics from DEG results

rawpval <- list("pval1"=All[["pvalue_1"]],"pval2"=All[["pvalue_2"]],

"pval3"=All[["pvalue_3"]],"pval4"=All[["pvalue_4"]],

"pval5"=All[["pvalue_5"]],"pval6"=All[["pvalue_6"]])

FC <- list("FC1"=All[["log2FoldChange_1"]],"FC2"=All[["log2FoldChange_2"]],

"FC3"=All[["log2FoldChange_3"]],"FC4"=All[["log2FoldChange_4"]],

"FC5"=All[["log2FoldChange_5"]],"FC6"=All[["log2FoldChange_6"]])

adjpval <- list("adjpval1"=All[["padj_1"]],"adjpval2"=All[["padj_2"]],

"adjpval3"=All[["padj_3"]],"adjpval4"=All[["padj_4"]],

"adjpval5"=All[["padj_5"]],"adjpval6"=All[["padj_6"]])

adjpval.logfc <- data.frame("adjpval1"=All[["padj_1"]],

"adjpval2"=All[["padj_2"]],

"adjpval3"=All[["padj_3"]],

"adjpval4"=All[["padj_4"]],

"adjpval5"=All[["padj_5"]],

"adjpval6"=All[["padj_6"]],

"logfc1"=All[["log2FoldChange_1"]],

"logfc2"=All[["log2FoldChange_2"]],

"logfc3"=All[["log2FoldChange_3"]],

"logfc4"=All[["log2FoldChange_4"]],

"logfc5"=All[["log2FoldChange_5"]],

"logfc6"=All[["log2FoldChange_6"]])

########## End of extract different statistics from DEG results

########## Meta analysis based on raw pvalue

library("metaRNASeq")

## fishercomb

fishcomb <- fishercomb(rawpval, BHth = 0.05)

hist(fishcomb$rawpval, breaks=100, col="grey", main="Fisher method", xlab = "Raw p-values (meta-analysis)")

##

## invnorm

invnormcomb <- invnorm(rawpval,nrep=c(3,3,3,3,3,3), BHth = 0.05)

hist(invnormcomb$rawpval, breaks=100, col="grey", main="Inverse normal method", xlab = "Raw p-values (meta-analysis)")

##

DEresults.All <- data.frame(All$ID, adjpval.logfc, fishcomb$adjpval,invnormcomb$adjpval)

write.csv(DEresults.All,"All_Results_metaRNASeq.csv")

########## End of meta analysis based on raw pvalue

################################################################# End of metaRNASeq
